# LncRNA LINC00662 promotes colon cancer tumor growth and metastasis by competitively binding with miR-340-5p to regulate CLDN8/IL22 co-expression and activating ERK signaling pathway

**DOI:** 10.1186/s13046-019-1510-7

**Published:** 2020-01-03

**Authors:** Bo Cheng, Aimei Rong, Quanbo Zhou, Wenlu Li

**Affiliations:** 1 Department of Emergency Surgery, The First Affiliated Hospital of Zhengzhou University, No. 1, Jianshe East Road, Zhengzhou City, 410008 Henan Province China; 2 Department of Gastroenterology, Zhengzhou Central Hospital Affiliated to Zhengzhou University, Zhengzhou City, 45000 Henan Province China; 3 Department of Anus and Intestine Surgery, The First Affiliated Hospital of Zhengzhou University, Zhengzhou City, 45000 Henan Province China; 4 Department of Stomatology, The First Affiliated Hospital of Zhengzhou University, Zhengzhou City, 45000 Henan Province China

**Keywords:** LncRNA LINC00662, Colon cancer, miR-340-5p, CLDN8, IL22, Growth, Metastasis

## Abstract

**Background:**

LncRNA LINC00662 is closely related to the occurrence and development of cancer. This study aims to explore the effect of LINC00662 on colon cancer tumor growth and metastasis and its molecular mechanism.

**Methods:**

CCK8, colony formation, transwell, scratch wound, TUNEL, flow cytometry, RT-PCR, western blotting and immunohistochemistry assays were used to detect the proliferation, apoptosis, invasion and migration of colon cancer cell and mRNA and protein expressions. Luciferase reporter and RNA pull down assays were used to detect the combination of LINC00662 and miR-340-5p or IL22 and the combination of miR-340-5p and CLDN8/IL22. Co-immunoprecipitation were used to detect the co-expression of CLDN8 and IL22 in colon cell lines. The targets of LINC00662 were predicated by Starbase v2.0. The target genes of miR-340-5p were predicated by miRDB and TargetScan. GO and KEGG enrichment analysis were performed by DAVID website.

**Results:**

LINC00662 was up-regulation in colon cancer tissues and cell lines. Univariate Cox regression analysis showed that the LINC00662 expression level was related to the poor prognosis. LINC00662-WT and miR-340-5p mimics co-transfection depressed luciferase activity and IL22/CLDN8-WT and miR-340-5p inhibitors co-transfection memorably motivated luciferase activity. LINC00662 overexpression promoted cell proliferation, invasion and migration, and inhibited cell apoptosis in colon cancer. In vivo xenograft studies in nude mice manifested that LINC00662 overexpression prominently accelerate tumor growth. There was an opposite reaction in the biological functions of colon cells and tumor growth between LINC00662 overexpression and LINC00662 inhibition in vitro and in vivo. The functions of miR-340-5p mimics regulating the biological functions of colon cells and tumor growth were consistent with those of LINC00662 inhibition. CLDN8 and IL22, as target genes of miR-340-5p, reversed the functions of LINC00662 affecting the biological functions of colon cells and the protein levels of Bax, Bcl-2, XIAP, VEGF, MMP-2, E-cadherin and N-cadherin. Co-immunoprecipitation experiments indicated that CLDN8 directly interact with IL22 in colon cell lines. LINC00662 regulated CLDN8 and IL22 expressions and the activation of ERK signaling pathway via targeting miR-340-5p.

**Conclusion:**

LINC00662 overexpression promoted the occurrence and development of colon cancer by competitively binding with miR-340-5p to regulate CLDN8/IL22 co-expression and activating ERK signaling pathway.

## Background

Colon cancer is a common malignant tumor of digestive tract in clinic, and its incidence and mortality are high [1]. With the adjustment of lifestyle and diet, the incidence of colon cancer is increasing year by year and becoming younger in China [2]. Like most malignant tumors, the pathogenesis of colon cancer is not entirely clear. At present, colon cancer is considered to be the combined effect of environmental factors and genetic factors. Studies have shown that the main factors affecting the incidence of colon cancer include environment, intestinal homeostasis, diet, alcohol and tobacco addiction and physical exercise [3]. Colon cancer treatment still is primarily surgical, chemotherapy and radiotherapy are supplementary. For therapeutic effect, there are significant individual differences among patients with colon cancer. In the patients with advanced colon cancer, the defects of the above therapy are obvious resulting in a poor prognosis. Postoperative metastasis for colon cancer chiefly includes hematological metastasis, peritoneal metastasis and distant lymph node metastasis, which are frequently accompanied by local recurrence [4]. Hematogenous metastasis is the dominating cause of failure in the treatment of colon cancer. The survival rate of colon cancer is overtly relevant to clinical stage, and the 5-year survival rates of patients with no metastasis, local metastasis and distant metastasis are 90, 70 and 10%, respectively [5]. Therefore, to find the markers of early diagnosis and explore the key molecules involved in the growth and metastasis of colon cancer is the focus of current research.

Long-stranded non-coding RNA is a class of RNA molecules whose transcriptional length exceeds that of 200 nt and can’t carry out coding proteins [6]. LncRNA usually is located in cytoplasm or nucleus. The number of lncRNA in the human genome is astonishingly large [7]. LncRNA participates in the regulatory processes of chromatin modification, transcriptional interference, transcriptional activation, nuclear transport, selective splicing and regulation of proto-oncogene activation, so as to regulate gene expression at epigenetic, transcriptional or post-transcriptional levels [8, 9]. Abnormal expression and functions of lncRNA are involved in the occurrence and development of many diseases, especially malignant tumors. It is reported in colon cancer that LINC01082 and lncRNA THOR can regulate cell proliferation, migration and invasion [10, 11] . LncRNA can not only directly participate in the post-transcriptional regulation of mRNA, including variable splicing, RNA editing, protein translation and transport, but also affect the expression of target genes by controlling microRNA [12]. In some tumor cells, lncRNA carries the seed sequence of miRNA to prevent miRNA from binding to its target mRNA. The functions of lncRNA AWPPH in proliferation of colon cancer cells were regulated by targeting GLUT-1 [13]; LncRNA CCAT1 promotes autophagy of liver cancer cells via regulating ATG7 by sponging miR-181 [12]; LncRNA HOTAIR promotes colon cancer progress by targeting miR-34a [14]. It is reported that the high expression of LINC00662 in lung cancer, gastric cancer and oral cancer promotes the occurrence and development of cancer [15, 16]. This suggests that LINC00662 is signally connected with cancer development. However, its role and related mechanisms in the initiation and progression of colon are unknown.

ERK, a serine/threonine protein kinase, is a signal transduction protein that transmits mitogen signals and is located in the cytoplasm. The activated ERK was transferred to the nucleus to regulate the activity of transcription factors and produce cellular effect. It is known that there are five subfamilies in the ERK family, including ERK1~ERK5. ERK1 and ERK2 are involved in regulating a series of physiological processes in different cells, including meiosis and mitosis. A variety of stimulators such as growth factors, cytokines, viruses, ligands of G protein-coupled receptors and oncogenes can activate ERK1 and ERK2 [17]. ERK signaling pathway is involved in the regulation of cell proliferation, differentiation and apoptosis. Activated ERK signaling pathway promotes the occurrence and development of a variety of cancers [18, 19]. mRNAs, miRNAs, lncRNAs and circRNAs affect the occurrence and development of cancer by regulating the activation of ERK signaling pathway. It is reported that lncRNA HOXD-AS1 influences the proliferation and invasion of hepatocellular carcinoma cells by regulating the activation of ERK signaling pathway [20]; miR-98 inhibits cell growth and invasion in retinoblastoma by targeting ERK signaling Pathway [21]; cicrRNA_006528 promotes the occurrence and development of breast cancer by activating ERK signaling pathway [22]; ectonucleoside triphosphate phosphohydrolase-7 (ENTPD7) inhibit the proliferation of lung cancer by inhibiting the activation of ERK signaling pathway [19]. Our previous studies have shown that CLDN8 promotes the proliferation and metastasis of colon cancer cells by activating MAPK/ERK signaling pathway [23]. Therefore, the role of LINC00662 in ERK signal pathway needs to be further studied.

## Methods

### Clinical samples

Cancer tissue and adjacent normal tissue in from 72 patients with colon cancer that resected in surgical procedures were collected from the First Affiliated Hospital of Zhengzhou University from July 2015 to July 2018. The patients’ clinical information is listed in Table 1. Liquid nitrogen was used for tissue store at − 80 °C. Each participant provided written informed consent. The use of human clinical tissues was approved by the Institutional Human Experiment and Ethics Committee of the First Affiliated Hospital of Zhengzhou University. All experiments were conducted under the rule of the Declaration of Helsinki.

**Table 1:** Relationship between LINC00662 expression and clinical parameters

Subgroup	Number	LINC00662 expression		χ^2^	*P*-value
		Low (*n* = 35)	High (*n* = 37)		
Age				0.001	0.974
< 60	41	20	21		
> =60	31	15	16		
Gender				0.259	0.611
male	31	14	17		
female	41	21	20		
Location				0.859	0.354
left	39	17	22		
right	33	18	15		
Histology				0.845	0.358
adenocarcinoma	41	18	23		
mucinous adenocarcinoma	31	17	14		
Stage				37.605	0.000
I + II	33	29	4		
III + IV	39	6	33		
T stage				8.354	0.004
Tis	41	26	15		
T1-T3	31	9	22		
N stage				16.068	0.000
N0	36	26	10		
N1 + N2	36	9	27		
M stage				22.363	0.000
M0	31	25	6		
M1	41	6	31		
Status					
death	12	2	10	5.882	0.015
alive	60	33	27		

### Cell line culture

Human colon epithelial cells (NCM460) and colon cancer cell lines (HCT116, HCT8, HCT29, LOVO, SW480, CT26 and LS174T) and were obtained from American Type Culture Collection (ATCC, Manassas, VA, USA). Modified RPMI-1640 medium (ThermoFisher) which is supplemented with 10% FBS including 100 μg/L penicillin and 100 μg/L streptomycin was applied to maintain all cells at 5% CO_2_ and 37 °C.

### Cell transfection

lncRNA LINC00662 pcDNA3.1 expression vector (5′-TCTACTTATATTTTATTCAAAAATTTA-3′), CLDN8 pcDNA3.1 expression vector (5′-ATGGCAACCCATGCCTTAGAAATCGCTG-3′), IL22 pcDNA3.1 expression vector (5′-ATGGCCGCCCTGCAGAAATCTGTGAGCTCTTT-3′) and empty vectors (pcDNA3.1-vector; 5′-ATCGCGCGTGTGCCGTGCAAAACTGCTACCAGT-3′) were designed and constructed by Sangon Biotech Co., Ltd. LINC00662 small interfering RNAs (siRNA; 5′- TAAATTTTGTAATAAAATATAAGTAGA-3′) and negative control (NC) siRNA were purchased form Thermo Fisher Scientific, Inc. miR-340-5p mimics (5′-TTATAAAGCAATGAGACTGATT-3′), miR-340-5p inhibitors (5′-AATCAGTCTCATTGCTTTATAA-3′), NC mimics (5′-TACTACGCATTATCCCATGCA-3′) and NC inhibitors (5′-TTAAACGTGTGTCGTACTGAA-3′) were obtained from Sigma-Aldrich (Merck KGaA, Darmstadt, Germany). Cell transfections were performed using Lipofectamine® 2000 reagent (Thermo Fisher Scientific, Inc.) at 37 °C with 10 nM of vectors, 40 nM of siRNAs and 40 nM of miRNAs. Cells were incubated with the transfection mixtures for 6 h. Cells treated with Lipofectamine® 2000 reagent only were used as untreated control cells. Cells transfected with empty vectors, NC siRNA, or NC miRNA were used as transfection controls. Cells transfected with pcDNA3.1-LINC00662, siRNA-LINC00662, siRNA-LINC00662 and CLDN8, siRNA-LINC00662 + IL22, miR-340-5p mimics and miR-340-5p inhibitors were collected 12 h after transfection prior to subsequent experimentation. Transfection efficiency was detected by RT-qPCR and western blot assays.

### Cell counting kit-8 (CCK8) assay

Cells transfected with pcDNA3.1-LINC00662, siRNA-LINC00662, siRNA-LINC00662 and CLDN8, siRNA-LINC00662 + IL22, miR-340-5p mimics and miR-340-5p inhibitors (2 × 10^4^ cells/mL) were incubated at 5% CO_2_ and 37 °C on 96-well plates (100 μL/well) for 24 h. CCK8 solution (Beyotime, Shanghai, China) was then added to each well after 24, 48, 72 and 96 h. Cell viability was estimated by a microplate reader which measure the absorbance values at a wavelength of 450 nm.

### Colony formation assay

Cells transfected with pcDNA3.1-LINC00662, siRNA-LINC00662, siRNA-LINC00662 and CLDN8, siRNA-LINC00662 + IL22, miR-340-5p mimics and miR-340-5p inhibitors were seeded into 12-well plate and incubated with complete medium at 37 °C for 14–21 days. Then, the cells were fixed with 4% paraformaldehyde and stained with 2% crystal violet. The images were obtained using an inverted microscope.

### Transwell assay

Cells (5 × 10^4^) were suspended in serum-free DMEM and added to chambers (8 mm, BD Biosciences) coated with BD BioCoat Matrigel. After incubation, the cells on the upper membrane surface were removed with a cotton tip. Then, crystal violet was used to stain them and then 5 representative microscopic fields were selected to count cells under an Olympus fluorescence microscope (Tokyo, Japan) to measure the rate of invasion. Experiments were conducted 3 times.

### Wound healing assay

In this study, cells in each group were implanted into 6-well culture plates with the density of 1.0 × 10^6^cells/well. After the cells had fused, a scratch was scraped with a pipette tip on the cell monolayer, and PBS (Beyotime, Wuhan, China) was subsequently applied to wash cells for 3 times, and FBS-free medium was used to seed cells. At 0 and 48 h incubation, the colon cancer cell lines were photographed using the inverted microscope (Olympus, Japan) and the scratch area was assessed using Image J software (National Institutes of Health, Bethesda, MD, USA). migration rate = migration distance/original distance.

### Flow cytometry

Cells grown normally without treatment were used as normal controls. Cell apoptosis was detected using Annexin V-fluorescein isothiocyanate (FITC)/propidium iodide (PI) apoptosis detection kit (Sigma) by flow cytometry. Cells (2 × 10^5^) were seeded into 6-well plates for 48 h. Subsequently, cells were washed with PBS and resuspended in binding buffer, followed by staining with 10 μL Annexin V-FITC for 10 min and 5 μL PI for 10 min in the dark according to the manufacturer’s instructions. The apoptotic (FITC positive and PI positive or negative) cells were analyzed by using a flow cytometer (Becton Dickinson, Franklin Lakes, NJ, USA).

### Target prediction and luciferase reporter assay

The putative targets of LINC00662 were predicted by the starbase v2.0. The reporter vector pmiRGLO- LINC00662-wild-type (LINC00662 WT) or miRGLO- LINC00662-mutant (LINC00662 MUT) containing the predicted miR-340-5p binding sites were purchased from GenePharma (Shanghai, China). LINC00662 WT or LINC00662 MUT was co-transfected with miR-340-5p mimics/inhibitors or NC mimics/inhibitors using Lipofectamine 2000 (invitrogen, USA). The putative targets of miR-340-5p were predicted by the TargetScan and miRDB. The reporter vector pmiRGLO- CLDN8-wild-type (CLDN8 WT) or miRGLO-CLDN8-mutant (CLDN8 MUT) and the reporter vector pmiRGLO-IL22-wild-type (IL22 WT) or miRGLO-IL22-mutant (IL22 MUT) containing the predicted miR-340-5p binding sites were purchased from GenePharma (Shanghai, China). CLDN8/IL22 WT or CLDN8/IL22 MUT was co-transfected with miR-340-5p mimics/inhibitors or NC mimics/inhibitors using Lipofectamine 2000 (invitrogen, USA). After 48 h, firefly and renilla luciferase activities were measured with the Dual Luciferase Reporter assay system (Promega, USA). The luciferase activities were normalized with the renilla luciferase activity.

The 5’UTR, coding sequences (CDS) and 3′UTR of IL22 were in- serted into PMIR-Reporter vector, denoted as Luc-IL22–5’UTR, Luc- IL22-CDS and Luc- IL22–3′UTR, respectively. The promoter sequences of IL22 were cloned into pGL3 vector, named as pGL3- IL22. For confirming the interaction between LINC00662 and IL22, Luc- IL22–5’UTR or Luc-S IL22-CDS or Luc- IL22–3′UTR or pGL3- IL22 was co-transfected with pcDNA3.1-LINC00662 infection in colon cancer cells (HCT29, LS174T, LOVO and CT26 cells). 72 h later, cells were lysed with Reporter lysis buffer (cat. no, E397 A; Promega Corporation, Madison, WI, USA) and luciferase activity was measured with VivoGlo Luciferin kit (cat. no., P1041; Promega Corporation) using a lumin- ometer (Thermo Fisher Scientific, Inc.) and normalized to β-gal activity.

### Biotin RNA pull-down assay

This assay was conducted as previously reported. Biotin-labeled sense or antisense oligos of LINC00662 were incubated with HCT29, LS174T, LOVO and CT26 cell lysate for 1 h. The complex was pull down by streptavidin-coupled agarose beads (Invitrogen). Sense probes included 5′-(biotin-) TGTGGAGATGGCTGGTACCAGT-GCAAGACG-3′, 5′-(biotin-) GGTACAGGACGCAACCAGA-GACGGGAGGTA-3′ and 5′-(biotin-)AGGTAGGAGTGCGG-TACAGGTACGGGCACC-3′. Antisense probes comprised 5′- (biotin-)CCTGAACCCTTGCCAGTATCCTGACCACGT-3′, 5′- (biotin-)ACCTCCTGTCCTAGGTCCTGCGTCCTTTCG-3′ and 5′-(biotin-) CCTACTGCGCTAGCCGGGTCCACCACTTCT-3′. The isolated RNA was transcribed into cDNA and then the amounts of LINC00662 and miR-340-5p were measured by RT-PCR as described in the method of RT-PCR.

### Functional enrichment analysis

The gene targets of miR-340-5p were identified using TargetScan and miRDB database. The Venn tool (Venn v2.0.2) was used to filter miRNA target genes into all three programs. Gene ontology (GO) categories (Biological Process, Cellular Components, and Molecular Processes) and the Kyoto Encyclopedia of Genes and Genomes (KEGG) pathway analysis (Arraystar Inc., Rockville, USA) were used to perform the functional analysis for predicted miRNA target genes.

### Immunohistochemistry

Immunohistochemistry was performed as described previously [24]. Briefly, the antibody against VEGF and MMP-2 (Proteintech) was tested on sections from a tumor tissue array. To quantify the status of VEGF and MMP-2 protein expression in those groups, the intensity of the VEGF and MMP-2 immunoreaction was scored as follows: 0, none; 1, weak; 2, moderate; and 3, intense.

### Xenograft tumor model

For tumor growth assay, HCT29 cells transfected with pcDNA3.1-vector or pcDNA3.1-LINC00662 and CT26 cells transfected with NC-siRNA or LINC00662-siRNA were trypsinized and washed and resuspended in DMEM without FBS. 20 male athymic nude mice (SLAC laboratory animal Center, Shanghai, China) were randomly divided into 4 groups (5 mice/group), and 2 × 10^6^ HCT29 or CT26 cells were subcutaneously injected into the right armpit of mice. The tumor size was determined every 3–4 days after tumor formed (around 1–2 weeks). At 30 days after injection, the mice were euthanized and the excised tumor tissues were formalin-fixed, paraffin-embedded, sectioned and then analyzed with VEGF and MMP-2 immunohistochemistry, and the tumors weight were weighted by a digital balance and tumor volume were measured by a ruler.

### Co-immunoprecipitation (co-IP)

Co-immunoprecipitation was performed as described previously [25]. Both the input and IP samples were analyzed by Western blot using various antibodies at the following dilutions: IL22 antibody (1:1000), CLDN8 antibody (1:1000), Flag-tag antibody (1:1000), HA-tag antibody (1:1000) and normal rabbit/mouse IgG (CST).

### Western-blot analysis

Total protein was extracted from cells. Proteins were separated by sodium dodecyl sulfate-polyacrylamide gel electrophoresis (SDS-PAGE) (Beyotime, Jiangsu, China) and then transferred onto PVDF membrane (Millipore, Billerica, MA, USA). The membranes were blocked with 5% skimmed at room temperature for 2 h. Anti-CLDN8, anti-IL22, anti-p-ERK, anti-ERK, anti-cleaved caspase-3, anti-bax, anti-bcl-2, anti-XIAP, anti-VEGF, anti-MMP-2, anti-E-cadherin, anti-N-cadherin and anti-GAPDH (1:800, abcam) were added overnight at 4 °C. The membranes were subsequently incubated with goat anti-rabbit IgG secondary antibody conjugated to horseradish peroxidase (1:5000, abcam) at room temperature for 2 h. Finally, proteins were visualized using a WestrenBright ECL Kit (Advansta, USA).

### RNA extraction and real-time PCR

The total RNA was extracted by using a TRIzol reagent. The first-strand cDNA was synthesized from 1 μg of total RNA using the Reverse Transcription System Bestar qPCR RT Kit according to the manufacturer instruction. Real-time PCR was carried out with an ABI 7500 Real-Time PCR System (Applied Biosystems, Lincoln Centre Drive, Foster City, CA 94404, USA). Each assay was performed in triplicate, and *β*-actin was used as the endogenous control gene. The relative amount of LINC00662, miR-340-5p, CLDN8 and IL22 were calculated using with a 2^−*ΔΔ*Ct^ method and normalized using GAPDH as an internal control. The primers used in this study were shown below: for miR-340-5p, 5′-CCGTTAGTTACGATTCGAAG-3′ (forward), 5′- AGGCCGCGCGTAGTGATGCAACA-3′ (reverse); for U6: 5′- AACCTTATATCGGGCGGGA-3′ (forward), 5′-TTACGGCGATGCATAAT-3′ (reverse); for LINC00662: 5′-CGGGCGATTATCGACGATC-3′ (forward), 5′- TCGGGATCGACTACCCTAGGTAC-3′ (reverse); for IL22: 5′- AATGGCGGGCTAGGGGCCCTT-3′(forward), 5′- CCTAGCTACGAATCCTAGGAGA-3′ (reverse); for CLDN8: 5′- ATATACGTGTGCGTACGT-3′(forward), 5′-CGGCGTAGCTGAACCCTGGTA-3′ (reverse); for GAPDH: 5′- CCTAGGTAAACTAGACGA-3′(forward), 5′- ATTATCTGTGTCTGCATGGC-3′ (reverse).

### Statistical analysis

All statistical analyses were conducted using statistical analysis software (IBM SPSS Software, Version 18.0). The relationship between the expression of miR-340-5p and LINC0062 was measured by Spearman rank correlation. In this study, we defined the relative LINC0062 expression > 4 as high expression and 4 is derived from the mean values of all samples. Survival analysis were compared using the univariate Cox proportional hazard model among different LINC0062 mRNA expression levels in colon cancer tissue. The correlations between LINC0062 expression and clinicopathological characteristics was examined by χ^2^ test. The results presented as mean ± SD were analyzed with a two-sided Student’s t-test for two groups and one-way ANOVA test for three or more groups. *P* < 0 .05 was considered as statistically significant. Survival curves were constructed using the Kaplan-Meier method and analyzed by the log-rank test.

## Results

### LINC00662 was highly expression in colon cancer tissues and cell lines and closely connected with OS

**Fig. 1:**
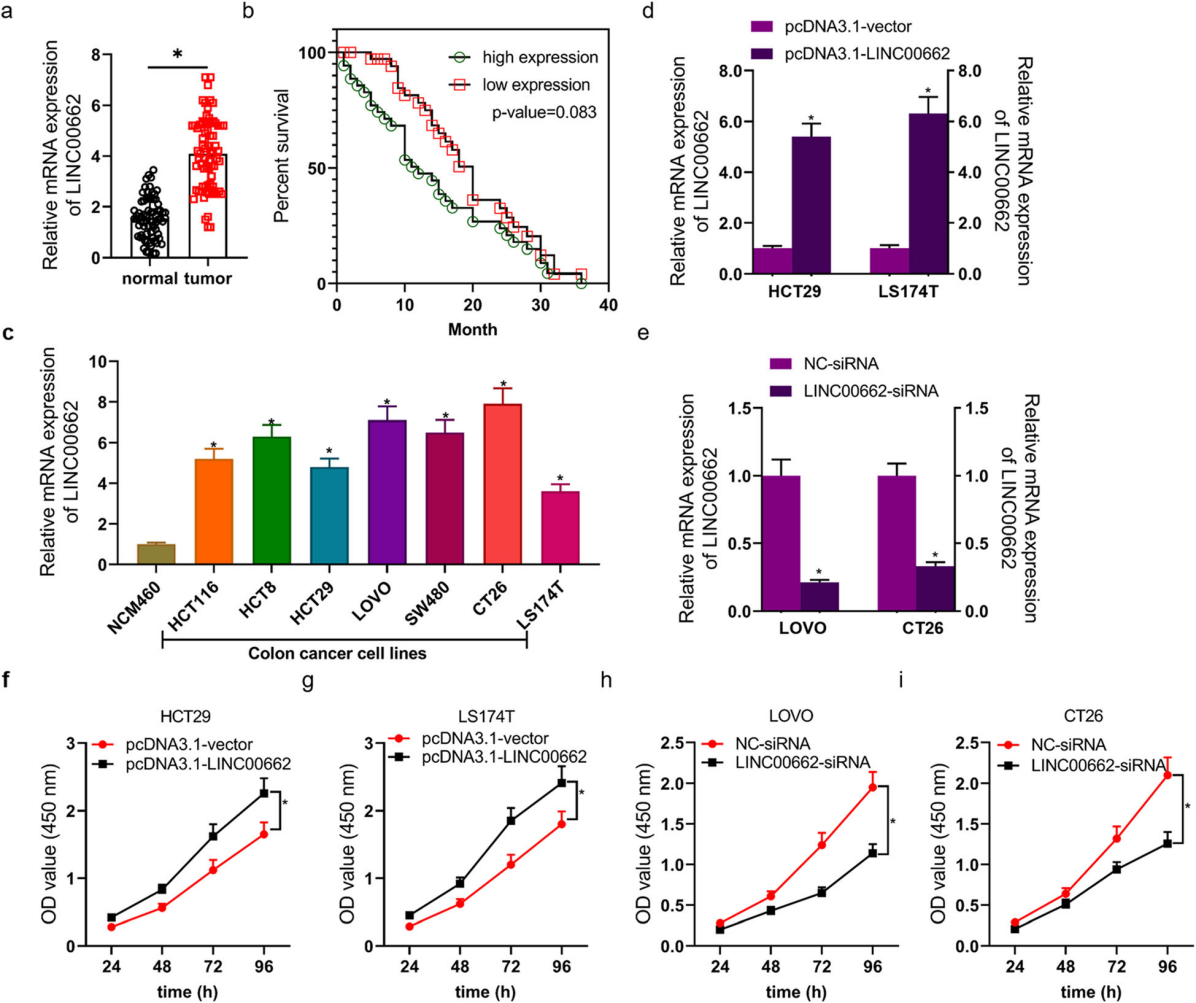
LINC00662 was highly expression in colon cancer tissues and cell lines and closely connected with OS and cell viability (**a**) RT-PCR assay was used to detected LINC00662 mRNA level in colon cancer tissues; (**b**) Kaplan-Meier analysis was used to showed survival rate of colon cancer patients with overexpression of LINC00662 and inhibition of LINC00662; (**c**) RT-PCR assay was used to detect LINC00662 mRNA level in colon cancer cell lines; (**d**) RT-PCR assay was used to detectLINC00662 mRNA level in LINC00662 overexpression plasmids transfected HCT29 and LS174T cells; (**e**) RT-PCR assay was used to detect LINC00662 mRNA level in LINC00662 knockdown plasmids transfected LOVO and CT26 cells; (**f** and **g**) CCK8 assay was used to detect cell viability of LINC00662 overexpression plasmids transfected HCT29 and LS174T cells; (**h** and **i**) CCK8 assay was used to detect cell viability of LINC00662 overexpression plasmids transfected LOVO and CT26 cells. GAPDH was used as a load control. Data are presented as the mean ± standard deviation. *P < 0.01 vs. normal group/ NCM460/pcDNA3.1-vector group/NC-siRNA group

LINC00662 was markedly high expression in tumor tissues from 72 patients with colon cancer (Fig. 1a). The survival rate of colon cancer’s patients with LINC00662 high expression was higher than that of colon cancer’s patients with LINC00662 low expression, whereas, there was no statistically significant difference in survival rate between LINC00662 high expression and LINC00662 low expression (the relative expression of LINC00662 > 4 as high expression; Fig. 1b). Following, the result revealed that LINC00662 expression was notably relevant to stage, TNM stage and survival status in colon cancer’s patients (Table 1). Univariate Cox regression was devoted to analyze the conceivable prognostic factors in colon cancer’s patients and uncovered that LINC00662 expression was memorably connected with overall survival (OS) (Table 2). At the same time, OS of colon cancer’s patients had not something to do with age, gender, location, histology, stage and TNM stage in univariate cox regression model. LINC00662 signally anabatic in colon cancer cell lines including HCT116, HCT8, HCT29, LOVO, SW480, CT26 and LS174T cells (Fig. 1c). After HCT29 and LS174T cells were transfected with empty vector or vector expressing LINC00662, LINC00662 was markedly ascending in LINC00662 overexpressed vector transfected HCT29 and LS174T cells (Fig. 1d). After LOVO and CT26 cells were transfected with siRNA-NC or siRNA-LINC00662, LINC00662 was prominently descending in siRNA-LINC00662 transfected LOVO and CT26 cells (Fig. 1e).

**Table 2:** Univariate Cox proportional hazards analyses of LINC00662 expression and overall survival for patients with colon cancer

	HR (95% CI)	*P*-value
LINC00662		
low vs high	0.35 (0.133–0.919)	0.033*
Age		
< 60 vs > =60	0.988 (0.55–1.775)	0.968
Gender		
male vs female	1.019 (0.577–1.798)	0.95
Location		
Left vs right	0.586 (0.322–1.065)	0.08
Histology		
Adenocarcinoma vs Mucinous adenocarcinoma	0.952 (0.539–1.681)	0.865
Stage		
I + II vs III + IV	1.825 (0.834–3.994)	0.132
T stage		
Tis vs T1-T3	1.734 (0.947–3.173)	0.074
N stage		
N0 vs N1 + N2	0.803 (0.44–1.464)	0.473
M stage		
M0 vs M1	1.011 (0.496–2.062)	0.975

*HR* Hazard ratio, *CI* Confidence interval. ^*^p < 0.05

### LINC00662 dramatically influenced the proliferation, apoptosis, invasion and migration of colon cancer cells

**Fig. 2:**
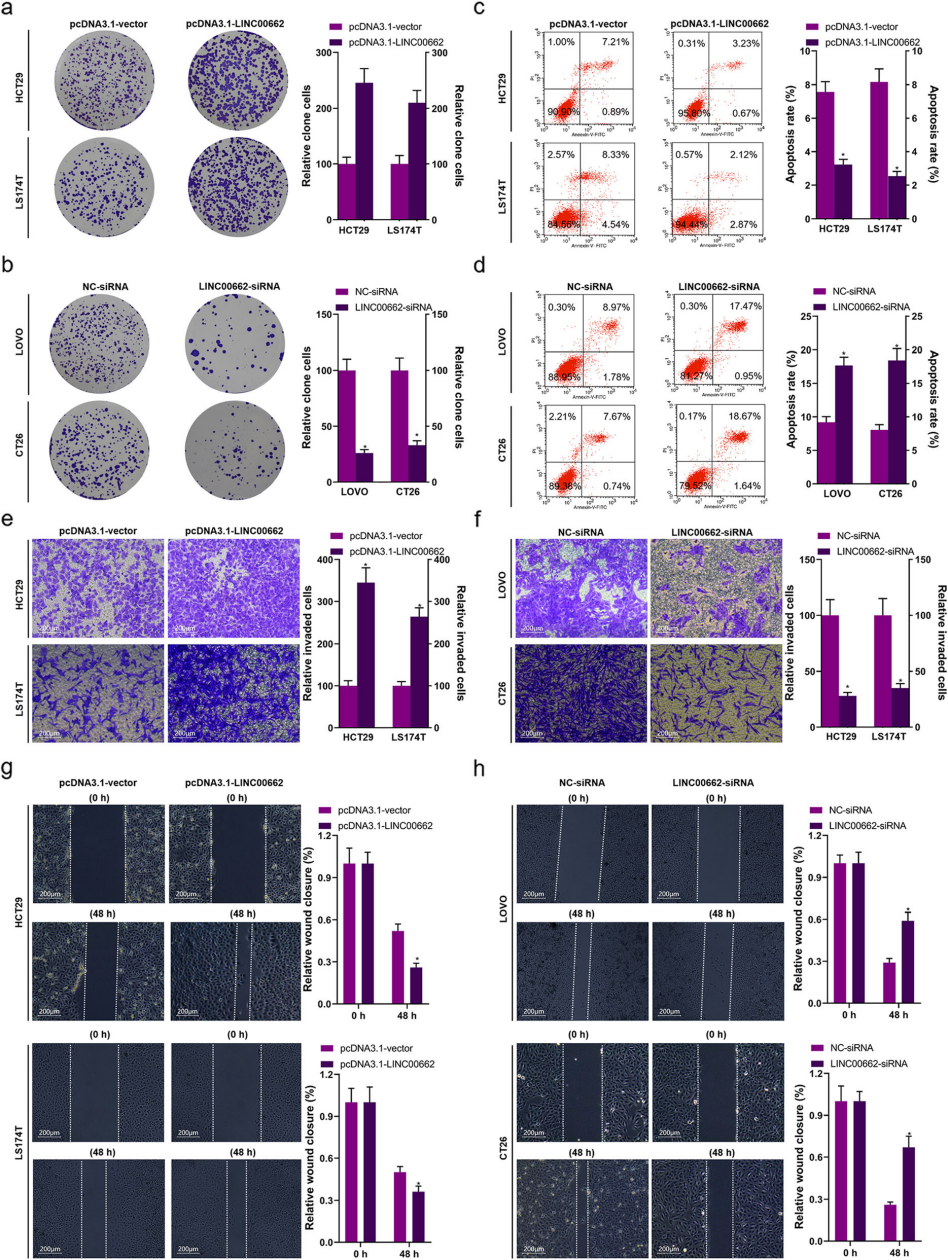
LINC00662 dramatically influenced the proliferation, apoptosis, invasion and migration of colon cancer cells (**a**) Clone formation assay was used to detect cell proliferation in LINC00662 overexpression plasmids transfected HCT29 and LS174T cells; (**b**) Clone formation assay was used to detect cell proliferation in LINC00662 knockdown plasmids transfected LOVO and CT26 cells; (**c**) Flow cytometry assay was used to detect cell apoptosis in LINC00662 overexpression plasmids transfected HCT29 and LS174T cells; (**d**) Flow cytometry assay was used to detect cell apoptosis in LINC00662 knockdown plasmids transfected LOVO and CT26 cells; (**e**) Transwell assay was used to detect cell invasion in LINC00662 overexpression plasmids transfected HCT29 and LS174T cells; (**f**) Transwell assay was used to detect cell invasion in LINC00662 knockdown plasmids transfected LOVO and CT26 cells; (**g**) Wound healing assay was used to detect cell migration in LINC00662 overexpression plasmids transfected HCT29 and LS174T cells; (**h**) Wound healing assay was used to detect cell migration in LINC00662 knockdown plasmids transfected LOVO and CT26 cells. Data are presented as the mean ± standard deviation. *P < 0.01 vs. pcDNA3.1-vector group/or NC-siRNA group

**Fig. 3:**
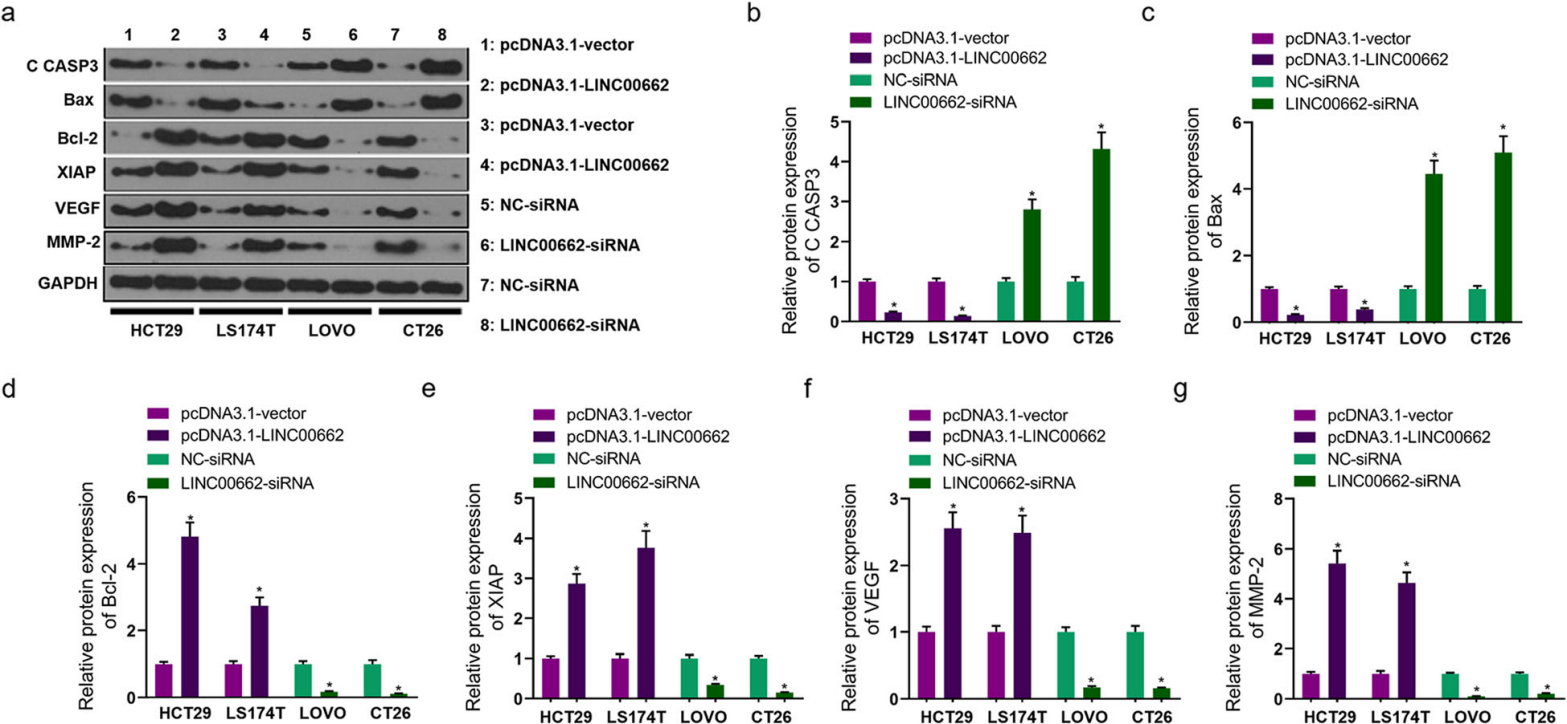
LINC00662 dramatically influenced the expressions of proliferation and apoptosis-related proteins and invasion and migration-related proteins In LINC00662 overexpression plasmids transfected HCT29 and LS174T cells and LINC00662 knockdown plasmids transfected LOVO and CT26 cells, (**a**) Western blot was used to detect cleaved-caspase-3, Bax, Bcl-2, XIAP, VEGF and MMP-2 protein levels, (**b**) Statistical graph of cleaved caspase-3 protein level, (**c**) Statistical graph of Bax protein level, (**d**) Statistical graph of Bcl-2 protein level, (**e**) Statistical graph of XIAP protein level, (**f**) Statistical graph of VEGF protein level and (**g**) Statistical graph of MMP-2 protein level. GAPDH was used as a load control. Data are presented as the mean ± standard deviation. *P < 0.01 vs. pcDNA3.1-vector group/NC-siRNA group

CCK8 and clone formation assays were utilized for confirming the proliferation of LINC00662 overexpression or LINC00662 inhibition transfected colon cancer cells. High expression of LINC00662 observably facilitated the viability of HCT29 and LS174T cells (Fig. 1f and g), in opposite terms, low expression of LINC00662 observably suppressed the viability of LOVO and CT26 cells (Fig. 1h and i). High expression of LINC00662 endowed HCT29 and LS174T cells with strong colony forming ability to increase cell proliferation (Fig. 2a), conversely, low expression of LINC00662 prominently depressed colony forming ability of LOVO and CT26 cells to reduce cell proliferation (Fig. 2b). Flow cytometry results had displayed that high expression of LINC00662 signally declined HCT29 and LS174T cells’ apoptosis (Fig. 2) and low expression of LINC00662 signally expedited LOVO and CT26’ apoptosis (Fig. 2d). By means of transwell assay, we found that the invasion ability of vector expressing LINC00662 transfected HCT29 and LS174T cells were markedly increased (Fig. 2e) and the invasion ability of siRNA-LINC00662 transfected LOVO and CT26 cells were markedly lowered (Fig. 2f). Next, the results of scratch-wound assay manifested that the migration ability of HCT29 and LS174T cells was observably inhibited by LINC00662 overexpression (Fig. 2g), otherwise, the migration ability of LOVO and CT26 cells was observably raised by LINC00662 inhibition (Fig. 2h). The apoptosis-related proteins including CASP3, Bax, Bcl-2 and XIAP, and the proliferation and metastasis-related proteins including VEGF and MMP-2 in protein level of colon cancer cells (HCT29, LS174T, LOVO and CT26 cells) transfected with LINC00662 overexpression or LINC00662 inhibition were detected by means of western blotting (Fig. 3a). The results uncovered that high expression of LINC00662 signally descended cleaved CASP3 expression and Bax expression of HCT29 and LS174T cells, and low expression of LINC00662 signally motivated cleaved CASP3 expression and Bax expression of LOVO and CT26 cells in protein level (Fig. 3b and c). Simultaneously, high expression of LINC00662 memorably facilitated the expressions of Bcl-2, XIAP, VEGF and MMP-2 in protein level of HCT29 and LS174T cells, and low expression of LINC00662 memorably descended the expressions of Bcl-2, XIAP, VEGF and MMP-2 in protein level of LOVO and CT26 cells (Fig. 3d, e, f and g).

### LINC00662 dramatically affected tumor growth of colon cancer in vivo

**Fig. 4:**
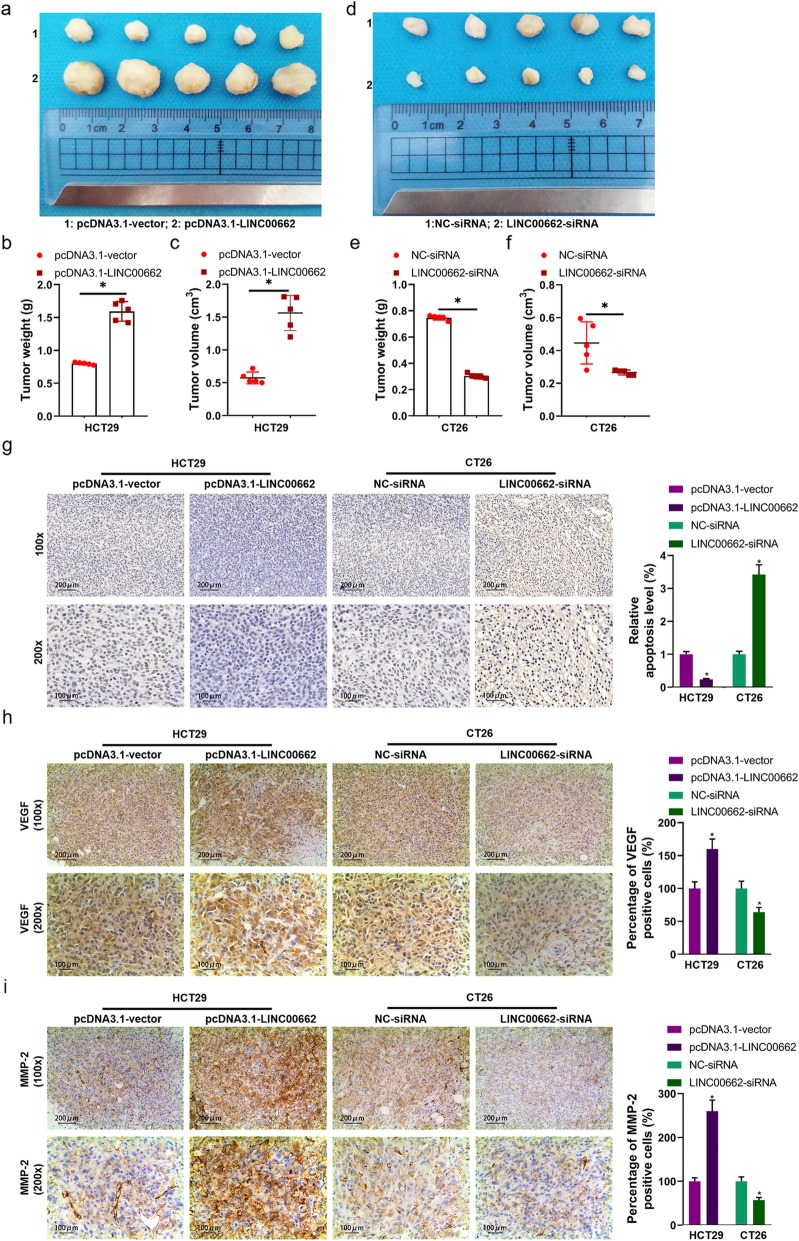
LINC00662 dramatically affected tumor growth of colon cancer in vivo (**a**) LINC00662 overexpression plasmids transfected HCT29 were injected with nude mouse; (**b**) Statistical graph of tumor weight in nude mouse carrying with LINC00662 overexpression plasmids transfected HCT29; (**c**) Statistical graph of tumor volume in nude mouse carrying with LINC00662 overexpression plasmids transfected HCT29; (**d**) LINC00662 knockdown plasmids transfected CT26 were injected with nude mouse; (**e**) Statistical graph of tumor weight in nude mouse carrying with LINC00662 knockdown plasmids transfected CT26; (**f**) Statistical graph of tumor volume in nude mouse carrying with LINC00662 knockdown plasmids transfected CT26; (**g**) TUNEL assay was used to detect cell apoptosis of tumor tissues carrying out LINC00662 overexpression plasmids transfected HCT29 and LINC00662 knockdown plasmids transfected CT26; (**h**) Immunohistochemistry assay was used to detect VEGF expression of tumor tissues carrying out LINC00662 overexpression plasmids transfected HCT29 and LINC00662 knockdown plasmids transfected CT26; (**i**) Immunohistochemistry assay was used to detect MMP-2 expression of tumor tissues carrying out LINC00662 overexpression plasmids transfected HCT29 and LINC00662 knockdown plasmids transfected CT26. Data are presented as the mean ± standard deviation. *P < 0.01 vs. pcDNA3.1-vector group/NC-siRNA group

The nude mouse tumorigenicity assay was applied to evaluate the effect of LINC00662 on tumor growth. After the vector expressing LINC00662 transfected HCT29 cells were injected into the nude mouse for 30 days (Fig. 4a), tumor weight and tumor volume were markedly ascending (Fig. 4b and c). In siRNA-LINC00662 transfected CT26 cells injection of the nude mouse (Fig. 4d), tumor weight and tumor volume were markedly depressed (Fig. 4e and f). The results of TUNEL assay displayed that cell apoptosis was prominently inhibited by high expression of LINC00662 and was observably expedited by low expression of LINC00662 in the nude mouse (Fig. 4g). Using immunohistochemistry technique, we found that VEGF positive cells was anabatic in tumor tissues of nude mouse carrying with the vector expressing LINC00662 transfected HCT29 cells and was declined in tumor tissues of nude mouse carrying with siRNA-LINC00662 transfected CT26 cells, compared to vector or siRNA group (Fig. 4h). Besides, tumor tissues of LINC00662 overexpression group had more positive expression of MMP-2 than vector group, whereas, tumor tissues of LINC00662 inhibition group had lower positive expression of MMP-2 than siNC group (Fig. 4i).

### miR-340-5p, as a target miRNA of LINC00662, regulated the proliferation, apoptosis, invasion and migration of colon cancer cells

**Fig. 5:**
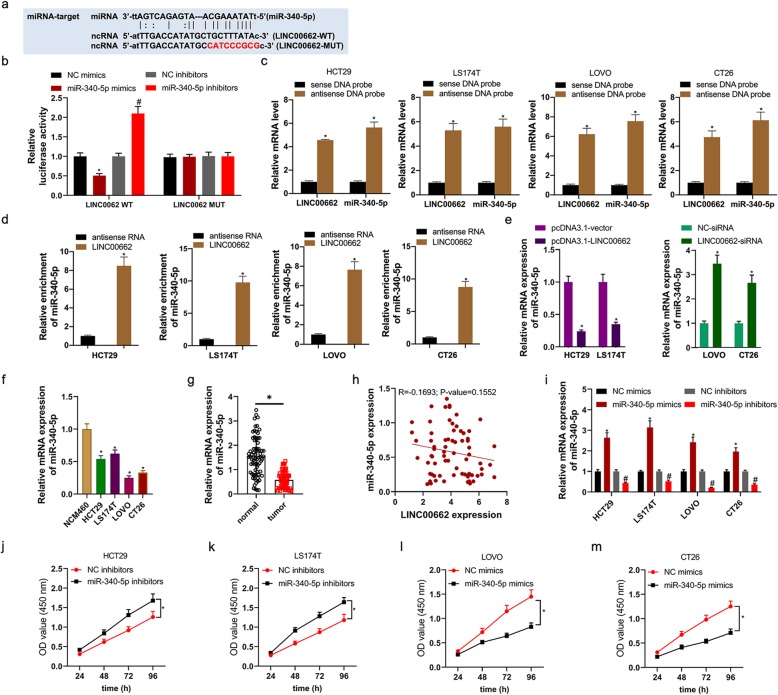
miR-340-5p, as a target miRNA of LINC00662, regulated the viability of colon cancer cells (**a**) Starbase v2.0 database showed that binding site of LINC00662 and miR-340-5p; (**b**) Luciferase reporter assays were used to prove that miR-340-5p can target LINC00662; (**c**) Biotin-coupled sense or antisense DNA probes targeting LINC00662 were incubated with HCT29, LS174T, LOVO, and CT26 cells lysate to pull down RNAs, followed by RT–PCR analysis of the amounts of LINC00202 and miR-3619-5p; (**d**) Biotin-labeled LINC00662 RNA and antisense RNA were incubated with HCT29, LS174T, LOVO, and CT26 cells lysate to pull down RNAs, and subsequently RT–PCR was performed to analyze the miR-340-5p amount; (**e**) miR-340-5p mRNA level was analyzed by RT-PCR in LINC00662 overexpression plasmids transfected HCT29 and LS174T cells; (**f**) miR-340-5p mRNA level was analyzed by RT-PCR in LINC00662 knockdown plasmids transfected LOVO and CT26 cells; (**g**) miR-340-5p mRNA level was analyzed by RT-PCR in colon cancer cell lines; (f) RT-PCR assay was used to detect miR-340-5p mRNA level in colon cancer tissues; (**h**) Correlation analysis of LINC00662 and miR-340-5p; (**i**) the transfection efficiency of miR-340-5p inhibitors was analyzed by RT-PCR in HCT29, LS174T, LOVO and CT26 cells; (**j** and **k**) CCK8 assay was used to detect cell viability of miR-340-5p inhibitors transfected HCT29 and LS174T cells; (**l** and **m**) CCK8 assay was used to detect cell viability of miR-340-5p mimics transfected LOVO and CT26 cells. GAPDH or U6 was used as a load control. Data are presented as the mean ± standard deviation. *P < 0.01 vs. NC inhibitors/sense DNA probe/antisense RNA and #*P* < 0.01 vs. NC mimics

**Fig. 6:**
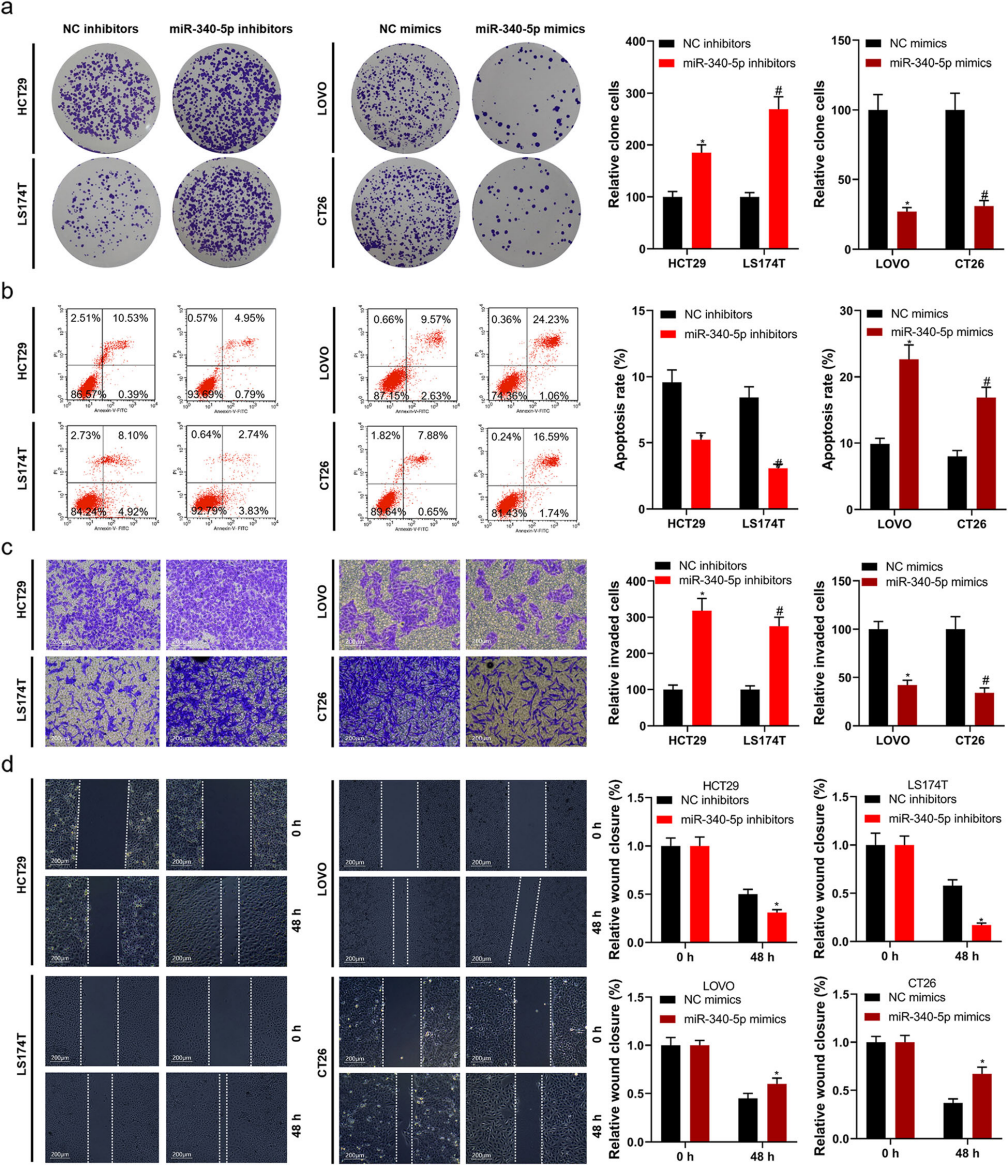
miR-340-5p dramatically influenced the proliferation, apoptosis, invasion and migration of colon cancer cells In miR-340-5p inhibitors/NC transfected HCT29 and LS174T cells and miR-340-5p mimics/NC transfected LOVO and CT26 cells, (**a**) Clone formation assay was used to detect cell proliferation, (**b**) Flow cytometry assay was used to detect cell apoptosis I, (**c**) Transwell assay was used to detect cell invasion, and (**d**) Wound healing assay was used to detect cell migration. Data are presented as the mean ± standard deviation. *P < 0.01 vs. NC inhibitors and #*P* < 0.01 vs. NC mimics

Starbase v2.0 database displayed that miR-340-5p had the putative binding site of LINC00662 (Fig. 5a). Luciferase reporter assay demonstrated that LINC00662-WT and miR-340-5p mimics co-transfection memorably depressed luciferase activity, however LINC00662-MUT and miR-370-3p mimics co-transfection failed to impact luciferase activity (Fig. 5b). Likewise, LINC00662-WT and miR-340-5p inhibitors co-transfection memorably motivated luciferase activity, however LINC00662-MUT and miR-370-3p inhibitors co-transfection failed to impact luciferase activity. Furthermore, LINC00662 antisense probe pull down not only LINC00662 RNA but also miR-340-5p (Fig. 5c). In addition, full-length LIN00662 RNA was able to enrich miR-340-5p from HCT29, LS174T, LOVO, and CT26 cells lysate (Fig. 5d). By means of RT-PCR assay, for HCT29 and LS174T cells, miR-340-5p expression in mRNA level was notably up-regulated by high expression of LINC00662, and for LOVO and CT26 cells, miR-340-5p expression in mRNA level was notably down-regulated by inhibition of LINC00662 (Fig. 5e). miR-340-5p expression in mRNA level was distinctly decreased in colon cancer tissues and cell lines (Fig. 5f and g). In mRNA level, miR-340-5p expression had a negative correlation with LINC00662 expression, however, *p*-value was more than 0.05 (Fig. 5h). By virtue of RT-PCR assay, miR-340-5p mimics/inhibitors/negative controls were favorably transfected into colon cancer cells including HCT29, LS174T, LOVO and CT26 cells (Fig. 5i). The results of CCK8 assay uncovered that miR-340-5p inhibitors visibly inhibited HCT29 and LS174T cells’ viability (Fig. 5j and k), and miR-340-5p mimics overtly facilitated LOVO and CT26 cells’ viability (Fig. 5l and m). We determined cell proliferation, apoptosis, invasion and migration with the method of colony formation, flow cytometry, transwell and scratch-wound. miR-340-5p inhibitors visibly motivated HCT29 and LS174T cells’ colony forming ability and miR-340-5p mimics distinctly inhibited LOVO and CT26 cells’ colony forming ability (Fig. 6a). Cell apoptosis was obviously reduced in miR-340-5p inhibitors transfected HCT29 and LS174T cells and was overtly expedited in miR-340-5p mimics transfected LOVO and CT26 cells (Fig. 6b). High expression of miR-340-5p was distinctly promoted the invasion and migration of HCT29 and LS174T cells and inhibition of miR-340-5p was visibly inhibited the invasion and migration of LOVO and CT26 cells (Fig. 6c and d).

### Enrichment analysis of the target genes of miR-340-5p

**Fig. 7:**
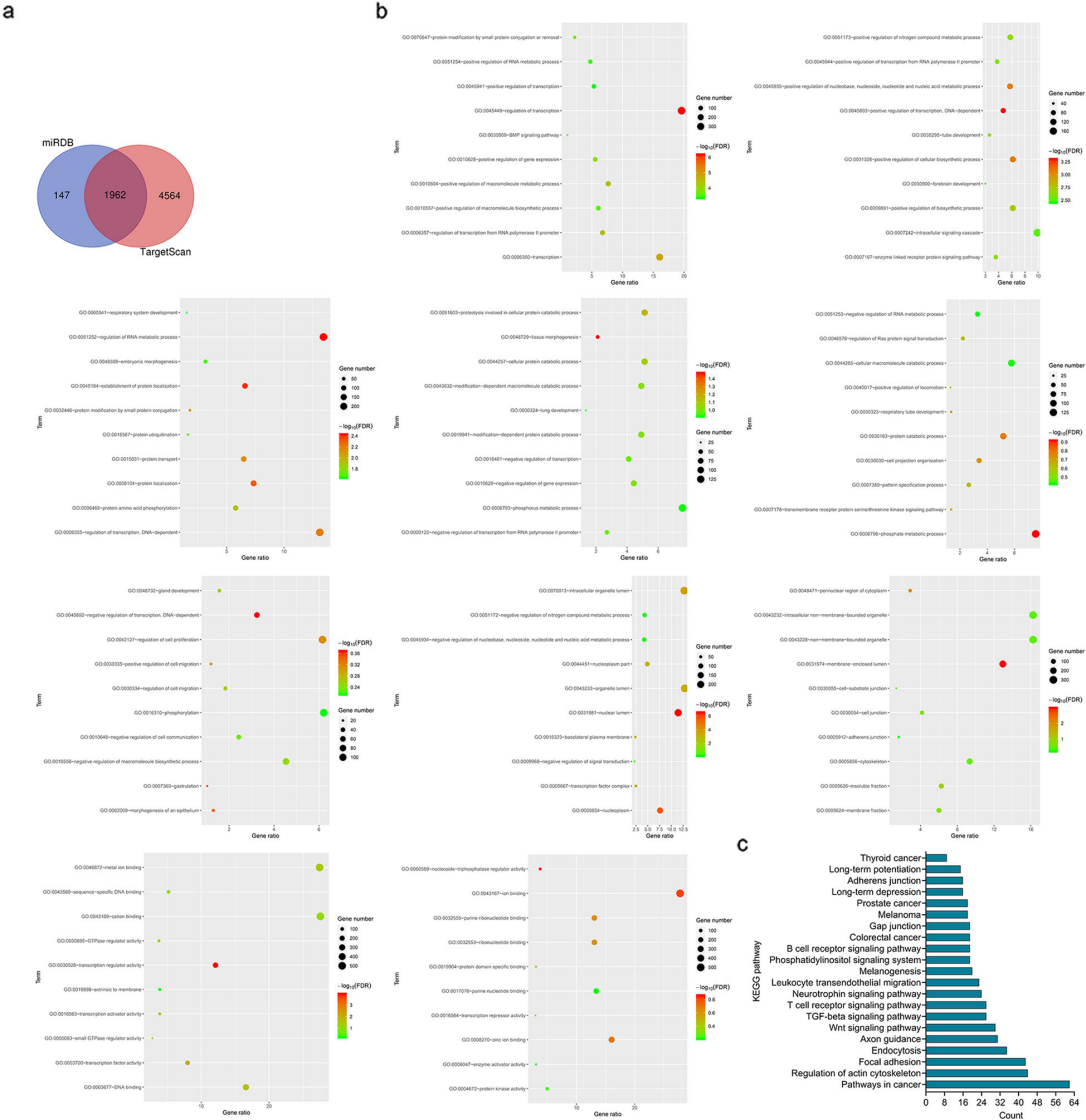
Prediction of target genes for miR-340-5p and functional enrichment analysis (**a**) 1962 target genes of miR-340-5p from miRDB and TargetScan database were shown in Venn diagram; (**b**) GO analysis showed that overlapping 1962 genes enriched in BP, CC and MF terms; (**c**) KEGG analysis was used to show the relationship of overlapping 1962 genes and pathways

2108 target genes of miR-340-5p were identified by miRDB database and 6525 target genes of miR-340-5p were identified by TargetScan database. A total of 1962 overlapping targets were identified by right of Venn tool (http://bioinformatics.psb.ugent.be/webtools/Venn/) (Fig. 7a). Base on DAVID database, 1962 targets distinctly enriched in BP term including ‘regulation of transcription’, ‘transcription’ and ‘regulation of transcription from RNA polymerase II promoter’, CC term including ‘nuclear lumen’, ‘nucleoplasm’ and ‘intracellular organelle lumen’ and MF term including ‘transcription regulator activity’, ‘transcription factor activity’ and ‘DNA binding’ (Fig. 7b). KEGG pathway analysis indicated that 1962 targets overtly enriched in ‘TGF-beta signaling pathway’, ‘Pathways in cancer’ and ‘Focal adhesion’ (Fig. 7c).

### LINC00662 regulated CLDN8/IL22 co-expression and the activation of ERK signaling pathway by competitively binding with miR-340-5p

**Fig. 8:**
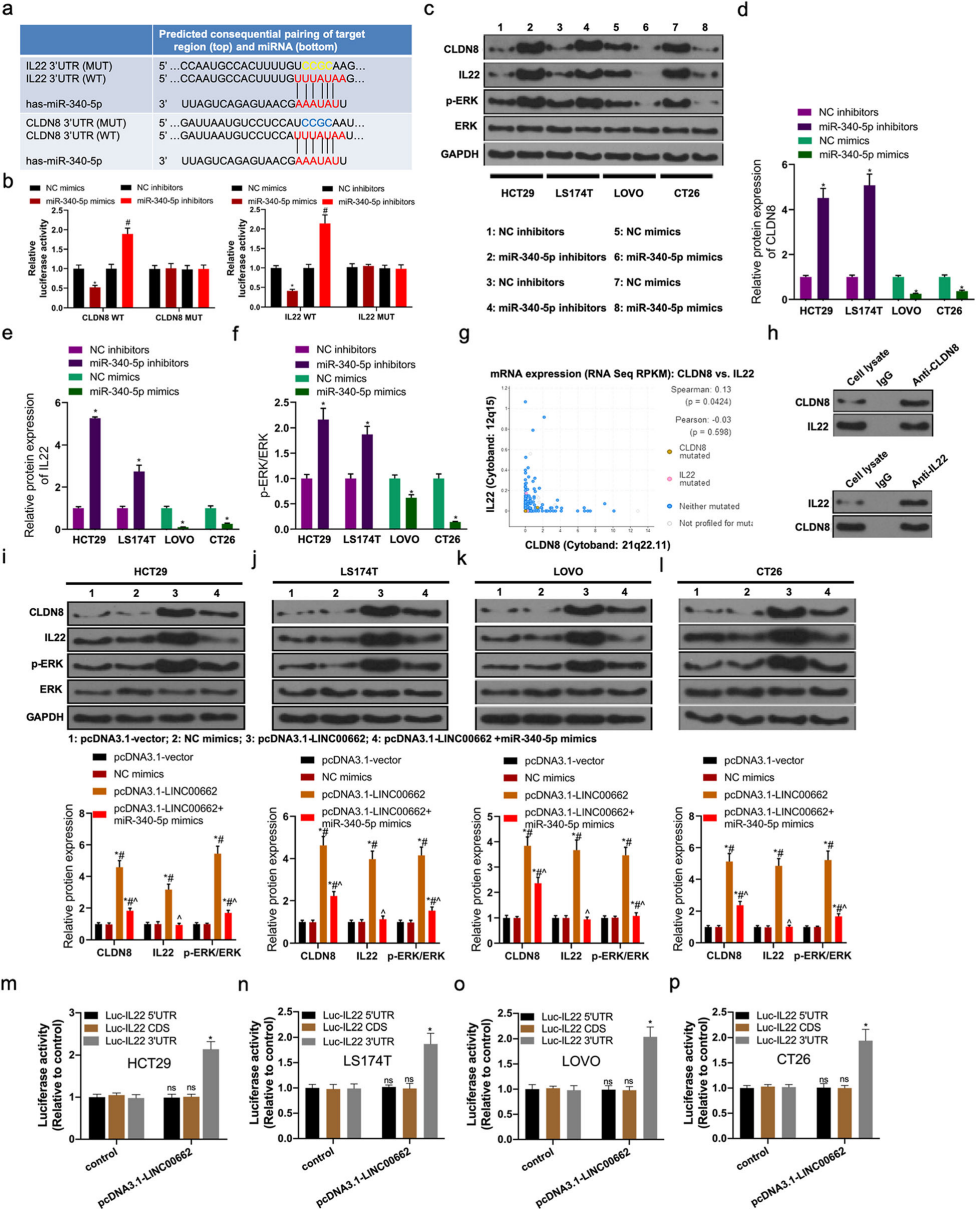
LINC00662 regulated CLDN8/IL22 co-expression and the activation of ERK signaling pathway by competitively binding with miR-340-5p (**a**) TargetScan database showed that binding site of CLDN8/or IL22 and miR-340-5p; (**b**) Luciferase reporter assays were used to prove that miR-340-5p can target CLDN8/or IL22; (**c**) Western blot was used to detect the expressions of CLDN8, IL22, p-ERK and ERK in protein levels in miR-340-5p inhibitors transfected HCT29 and LS174T cells and miR-340-5p mimics transfected LOVO and CT26 cells; (**d**) Statistical graph of CLDN8 protein level; (**e**) Statistical graph of IL22 protein level; (**f**) Statistical graph of p-ERK/ERK level; (**g**) cBioPrortal database showed co-expression relationship of CLDN8 and IL22 genes; (**h**) Co-immunoprecipitation experiments showed that CLDN8 directly interact with IL22 in colon cell lines; After LINC00662 overexpression plasmids and miR-340-5p mimics were transfected into HCT29 and LS174T cells, (**i** and **j**) Western blot was used to detect the expressions of CLDN8, IL22, p-ERK and ERK in protein levels; After LINC00662 knockdown plasmids and miR-340-5p mimics were transfected into LOVO and CT26 cells, (**k** and **l**) Western blot was used to detect the expressions of CLDN8, IL22, p-ERK and ERK in protein levels; (**m**-**p**) Luciferase activity of vector containing different region of IL22 was measured in cells with LINC00662 overexpression in colon cancer cell lines (HCT29, LS174T, LOVO and CT26 cells). GAPDH was used as a load control. Data are presented as the mean ± standard deviation. *P < 0.01 vs. NC inhibitors and #*P* < 0.01 vs. NC mimics

IL22 and CLDN8, as the target genes of miR-340-5p, were identified by TargetScan and miRDB database. IL22 and CLDN8 had the putative binding site of miR-340-5p (Fig. 8a). Luciferase reporter assay demonstrated that CLDN8-WT and miR-340-5p mimics co-transfection memorably depressed luciferase activity, however CLDN8-MUT and miR-340-5p mimics co-transfection failed to impact luciferase activity (Fig. 8b). Likewise, IL22-WT and miR-340-5p inhibitors co-transfection memorably motivated luciferase activity, however IL22-MUT and miR-370-3p inhibitors co-transfection failed to impact luciferase activity (Fig. 8b). In HCT29 and LS174T, miR-340-5p inhibitors overtly elevated the expressions of CLDN8, IL22 and phosphorylation (phosph)-ERK in protein level by virtue of western blotting. In LOVO and CT29 cells, miR-340-5p mimics distinctly declined the expressions of CLDN8, IL22 and phosph-ERK in protein level by virtue of western blotting (Fig. 8c, d, e and f). The CLDN8/IL22 gene co-expression relationship was verified using cBioPrortal database (http://www.cbioportal.org/) (Fig. 8g). Co-immunoprecipitation experiments indicated that CLDN8 directly interact with IL22 in colon cell lines (Fig. 8h). Next, high expression of LINC00662 prominently elevated the expressions of CLDN8, IL22 and phosph-ERK in protein level of colon cancer cells including HCT29, LS174T, LOVO and CT26 cells, however, the expressions of CLDN8, IL22 and phosph-ERK in protein level of LINC00662 overexpression transfected colon cancer cells were inhibited by miR-340-5p overexpression (Fig. 8i-l). To further clarify the interaction between LINC00662 and IL22 3′UTR. Luciferase re- porter vectors containing different regions of IL22 mRNA were co- transfected with pcDNA3.1-LINC00662 infection in HCT29, LS174T, LOVO, and CT26 cells. As shown in Fig. 8m-p, the luciferase activity of Luc-IL22–3′UTR was enhanced in colon cancer cells with LINC00662 over-expression, but the luciferase activity of Luc-IL22–5’UTR or Luc-IL22- CDS was unaffected.

### LINC00662 regulated the proliferation, apoptosis, invasion and migration of colon cancer cells by targeting CLDN8 and IL22

**Fig. 9:**
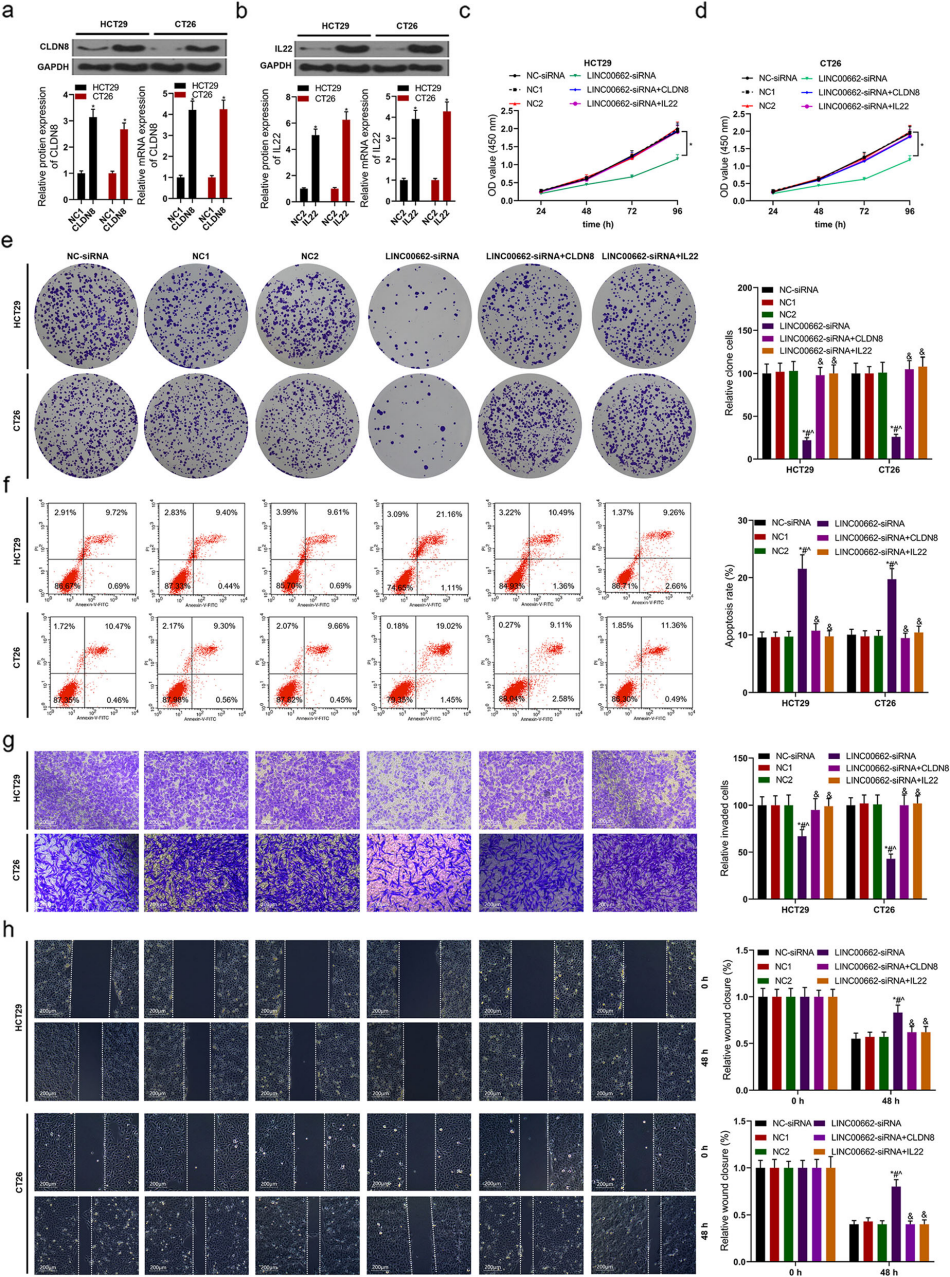
LINC00662 regulated the proliferation, apoptosis, invasion and migration of colon cancer cells by targeting CLDN8 and IL22 (**a**) Western blot and RT-PCR assays were used to detect CLDN8 expression in protein and mRNA levels in CLDN8 overexpression plasmids transfected HCT29 and CT26 cells; (**b**) Western blot and RT-PCR assays were used to detect IL22 expression in protein and mRNA levels in IL22 overexpression plasmids transfected HCT29 and CT26 cells; After LINC00662 knockdown plasmids and CLDN8/or IL22 overexpression plasmids were transfected into HCT29 and CT26 cell, (**c** and **d**) CCK8 assay was used to detect cell viability, (**e**) Clone formation assay was used to detect cell proliferation, (**f**) Flow cytometry assay was used to detect cell apoptosis, (**g**) Transwell assay was used to detect cell invasion, (**h**) Wound healing assay was used to detect cell migration. GAPDH was used as a load control. Data are presented as the mean ± standard deviation. *P < 0.01 vs. NC-siRNA group, #*P* < 0.01 vs. NC1 group, ^*P* < 0.01 vs. NC2 group and &*P* < 0.01 vs. LINC00662-siRNA group

According to the results of RT-PCR and western blot assays, the expressions of CLDN8 and IL22 in mRNA and protein levels of HCT29 and CT26 cells were up-regulated by CLDN8 overexpression and IL22 overexpression (Fig. 9a and b). By means of CCK8 and colon formation assays, in HCT29 and CT26 cells, the functions of LINC00662 knockdown declining cell viability and colony forming ability were visibly reversed by CLDN8 or IL22 overexpression (Fig. 9c-e). After siRNA-LINC00662 transfected HCT29 and CT26 cells were transfected with CLDN8 or IL22 overexpression, cell apoptosis was lower than that of LINC00662 knockdown group via flow cytometry assay (Fig. 9f). There was no significant difference in cell invasion and migration of HCT29 and CT26 cells among three groups including siRNA-NC, NC1 and NC2 groups. The functions of LINC00662 knockdown declining cell invasion and migration were abrogated by CLDN8 or IL22 overexpression in HCT29 and CT26 cells (Fig. 9g and h).

### LINC00662 regulated the activation of ERK signaling pathway by targeting CLDN8 and IL22

**Fig. 10:**
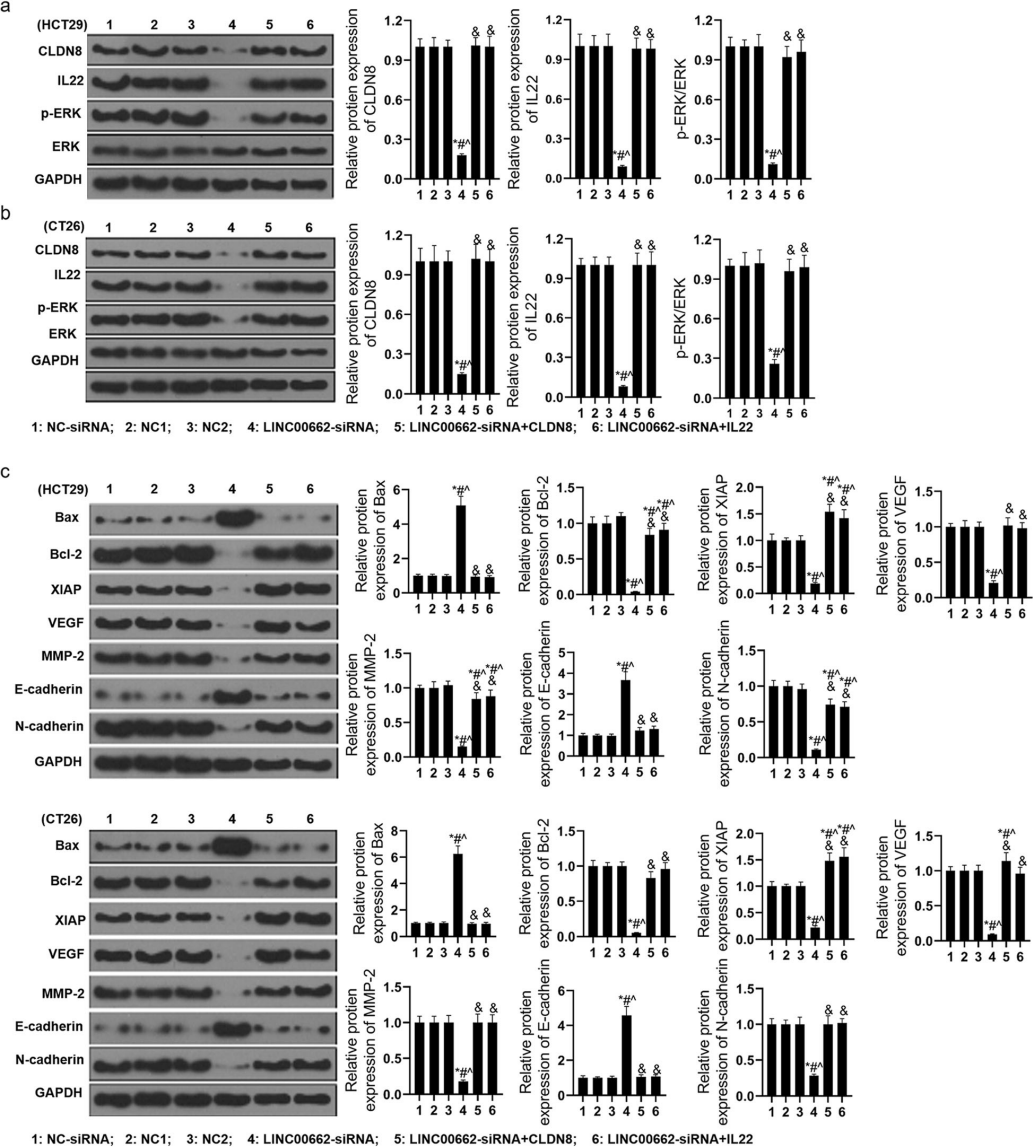
LINC00662 regulated the activation of ERK signaling pathway by targeting CLDN8 and IL22 (**a**) After LINC00662 knockdown plasmids and CLDN8/or IL22 overexpression plasmids were transfected into HCT29 cell, western blot assay was used to detect the expressions of CLDN8, IL22, p-ERK and ERK in protein levels; (**b**) After LINC00662 knockdown plasmids and CLDN8/or IL22 overexpression plasmids were transfected into CT26 cell, western blot assay was used to detect the expressions of CLDN8, IL22, p-ERK and ERK in protein levels; (**c**) After LINC00662 knockdown plasmids and CLDN8/or IL22 overexpression plasmids were transfected into HCT29 cell, western blot assay was used to detect the expressions of Bax, Bcl-2, XIAP, VEGF, MMP-2, E-cadherin and N-cadherin in protein levels; (**d**) After LINC00662 knockdown plasmids and CLDN8/or IL22 overexpression plasmids were transfected into CT26 cell, western blot assay was used to detect the expressions of Bax, Bcl-2, XIAP, VEGF, MMP-2, E-cadherin and N-cadherin in protein levels. GAPDH was used as a load control. Data are presented as the mean ± standard deviation. *P < 0.01 vs. NC-siRNA group, #*P* < 0.01 vs. NC1 group, ^*P* < 0.01 vs. NC2 group and &*P* < 0.01 vs. LINC00662-siRNA group

With the means of western blotting, the expression of CLDN8, IL22, phosph-ERK, Bax, Bcl-2, XIAP, VEGF, MMP-2, E-cadherin and N-cadherin in protein level of siRNA-LINC00662 and CLDN8/or IL22 overexpression co-transfected HCT29 and CT26 cells. The functions of LINC00662 knockdown declining the expression of CLDN8, IL22 and phosph-ERK in protein level of HCT29 and CT26 cells were reversed by CLDN8 or IL22 overexpression (Fig. 10a and b). The expression of Bax and E-cadherin in protein level of HCT29 and CT26 cells were distinctly increased by siRNA-LINC00662 and the expression of Bcl-2, XIAP, VEGF, MMP-2 and N-cadherin in protein level of HCT29 and CT26 cells were distinctly decreased by siRNA-LINC00662. Next, the functions of LINC00662 knockdown regulating Bax, Bcl-2, XIAP, VEGF, MMP-2, E-cadherin and N-cadherin in protein level of HCT29 and CT26 cells were reversed by CLDN8 or IL22 overexpression (Fig. 10c and d).

## Discussion

LncRNA plays an important role in the occurrence and development of cancer [6, 8, 11, 15]. In this study, high expression of LINC00662 was detected in colon cancer tissues and cell lines, and the survival rate of colon cancer patients with high expression of LINC00662 was lower than that of patients with low expression of LINC00662. Cox model analysis further showed that the expression of LINC00662 was significantly correlated with OS. This suggests that LINC00662 may play a role in the occurrence and development of colon cancer. The expression of LINC00662 was relatively low in HCT29 and LS174T cells, while LINC00662 was relatively high in LOVO and CT26 cells. Therefore, HCT29 and LS174T cells were used in cell transfection of LINC00662 overexpression, LOVO and CT26 cells were used in cell transfection of LINC00662 knockdown (Fig. 1). Cell proliferation, apoptosis, migration and invasion are the basic biological functions of tumor cells for tumor growth and metastasis [8]. The following results showed that in HCT29 and LS174T cells, high expression of LINC00662 prominently elevated cell vitality, clone formation ability, cell migration and invasion, and memorably declined apoptosis. On the contrary, LINC00662 knockout distinctly depressed cell activity. Clone forming ability, cell migration and invasion, overtly motivated cell apoptosis (Fig. 2). These results manifest that LINC00662 can visibly regulate the biological function of colon cancer cells in vitro. As a star molecule in the caspase family, Caspase-3 participates in the regulation of apoptosis, and its activity can be inhibited by XIAP [26]. Bcl-2 and Bax belongs to the bcl-2 family, which is not only involved in regulating the activity of caspase-3, but also can be regarded as he substrate of caspase-3 to act directly on the downstream genes of caspase-3 [26]. Therefore, caspase family and bcl-2 family are not only related to each other but also control each other in the process of apoptosis transmission and play a role in regulating apoptosis in a variety of cancer cells. Our results showed that high expression of LINC00662 notably reduced the expression of pro-apoptotic protein (caspase-3 and Bax) and promoted the expression of anti-apoptotic protein (bcl-2 and XIAP). On the contrary, LINC00662 knockout dramatically up-regulated the expression of pro-apoptotic protein (caspase-3 and Bax). The expression of anti-apoptotic protein (bcl-2 and XIAP) was descending. VEGF is a powerful cytokine that can produce a variety of biological effects. It can specifically act on vascular endothelial cells, induce vascular endothelial cell proliferation, and then promote tumor growth [27]. Therefore, VEGF is considered to be a marker of cell proliferation. Matrix metalloproteinases (MMPs) can promote tumor metastasis by degradation of extracellular matrix and basement membrane [28]. It is reported that MMP2 knockout can inhibit tumor metastasis [29]. In this study, LINC00662 overexpression distinctly expedited the protein levels of VEGF and MMP2, and LINC00662 knockout significantly inhibited the protein levels of VEGF and MMP2 in colon cancer cells (Fig. 3). Tumor formation experiment in nude mice further confirmed that LINC00662 significantly regulated tumor growth and metastasis (Fig. 4). To sum up, LINC00662 affects the biological function of colon cancer cells by regulating the expression of proliferation and apoptosis-related proteins and the expression of migration and invasion-related proteins in vivo and in vitro.

It is reported that overexpressed miR-340-5p signally inhibit the proliferation and invasion of lung cancer cells 18. However, the role and mechanism of miR-340-5p in colon cancer is unknown. Based on the starbase v2.0 database, we predict that miR-340-5p contains LINC00662 binding sites. Co-transfection of LINC00662-WT and miR-340-5pmimcs markedly inhibited the relative luciferase activity, while co-transfection of LINC00662-WT and miR-340-5p inhibitors obviously increased the relative luciferase activity. In addition, after HCT29 and LS174T cells were transfected with LINC00662 overexpression, miR-340-5p expression in mRNA was significantly decreased. After LOVO and CT26 cells were transfected with siRNA-LINC00662, miR-340-5p expression in mRNA was significantly increased. Further results showed that miR-340-5p was significantly down-regulated in colon cancer tissues and cell lines. There was a negative correlation between miR-340-5pexpression and LINC00662expression in mRNA level. The results of functional experiments show that the functions of miR-340-5p regulating cell proliferation, apoptosis, invasion and migration were coincidence with that of LINC00662 overexpression (Fig. 5 and Fig. 6). miRNA affects cellular biological function by targeting its target genes. We predicted the target gene of miR-340-5p by miRDB and TargetScan database. GO and KEGG enrichment analysis were used to predict the biological functions and pathways of 1962 target genes from miRDB and TargetScan databases (Fig. 7). Our previous study found that CLDN8 was overtly up-regulation in colon cancer tissues and cell lines, promoting cell proliferation, migration and invasion by activating MAPK/ERK signaling pathway [23]. Both IL22 and CLDN8 are target genes of miR-340-5p and are co-expressed in colon cancer cells. Through western blot assay, we found that the high expression of LINC00662 significantly increased the expression of CLDN8 and IL22 in protein level and activated the ERK signaling pathway in colon cancer cells. Combined with the previous results, the effects of LINC00662 overexpression and CLDN8 overexpression on the biological function of colon cancer cells were consistent. However, the high expression of LINC00662 up-regulated the protein levels of CLDN8 and IL22 in colon cancer cells and activated ERK signaling pathway were markedly reversed by miR-340-5p overexpression (Fig. 8). It was uncovered that LINC00662 regulated CLDN8/IL22 co-expression and the activation of ERK signaling pathway by competitively binding with miR-340-5p. The results of rescue experiment indicated that the functions of LINC00662 knockdown inhibiting cell proliferation, invasion and migration and promoting cell apoptosis were reversed by CLDN8 or IL22 overexpression (Fig. 9). Meanwhile the functions of LINC00662 knockdown inhibiting CLDN8, IL22 protein levels were reversed by CLDN8 or IL22 overexpression. The functions of LINC00662 inhibition inhibiting the activation of ERK signaling pathway was counteracted by CLDN8 or IL22 overexpression. It is reported that the activated ERK signaling pathway promotes the occurrence and development of cancer by up-regulating the expression of anti-apoptotic proteins, proliferation-related proteins and migration and invasion-related proteins. CLDN8 or IL22 overexpression reversed the effects of LINC00662 on the expression of Bax, Bcl-2, XIAP, VEGF, MMP-2, E-cadherin and N-cadherin in protein level (Fig. 10). Previous studies have shown that highly expressed LINC00662 activates ERK signaling pathway, and highly expressed CLDN8 can also activate ERK signaling pathway. miR-340-5p can target LINC00662 and CLDN8/IL22. Therefore, LINC00662 knockdown inhibited CLDN8/IL22 co-expression to inhibit the activation of ERK signaling pathway by competitively binding with miR-340-5p.

## Conclusions

In conclusion, high expression of LINC00662 promotes the occurrence and development of colon cancer by activating ERK signaling pathway. High expression of miR-340-5p can inhibit the proliferation, migration and invasion of colon cancer cells, and promote apoptosis. The role of highly expressed LINC00662 in promoting the occurrence and development of colon cancer by activating ERK signaling pathway is inhibited by miR-340-5p mimics. INC00662 may be a potential proto-oncogene by regulating the co-expression of CLDN8/IL22 through competitively binding to miR-340-5p to affect the occurrence and development of colon cancer.

## Data Availability

The datasets generated/analyzed during the current study are available.

## Abbreviations

Bax: BCL2 associated X, apoptosis regulator
Bcl-2: BCL2 apoptosis regulator
DAVID: Database for annotation visualization and integrated discovery
GO: Gene ontology
KEGG: Kyoto encyclopedia of genes and genomes
MMP-2: Matrix metallopeptidase 2
VEGF: Vascular endothlial growth factor
XIAP: X-linked inhibitor of apoptosis
